# Forearm muscles in the black lion tamarin (*Leontopithecus chrysopygus*)

**DOI:** 10.29374/2527-2179.bjvm002925

**Published:** 2025-09-08

**Authors:** Erick Candiota Souza, Micaela Ramos do Amaral, Daniel Marques Leopoldino Guerra, Marcelo Abidu-Figueiredo, Alcides Pissinatti, Vanessa Barbisan Fortes, Paulo de Souza

**Affiliations:** 1 Programa de Pós-graduação em Ciência Animal (PPGCA), Universidade Federal do Pampa (UNIPAMPA), Uruguaiana, RS, Brazil.; 2 Universidade Federal do Pampa (UNIPAMPA), Uruguaiana, RS, Brazil.; 3 Instituto de Ciências Biológicas e da Saúde, Universidade Federal Rural do Rio de Janeiro (UFRRJ), Seropédica, RJ, Brazil.; 4 Centro de Primatologia do Rio de Janeiro (CPRJ), Guapimirim, RJ, Brazil; Centro Universitário Serra dos Órgãos (UNIFESO), Teresópolis, RJ, Brazil.; 5 Departamento de Zootecnia e Ciências Biológicas, Universidade Federal de Santa Maria (UFSM), Palmeira das Missões, RS, Brazil.; 6 Programa de Pós-graduação em Ciência Animal (PPGCA), Universidade Federal do Pampa (UNIPAMPA), Uruguaiana, RS, Brazil.

**Keywords:** animal anatomy, thoracic limb, black lion tamarin, myology, primates, anatomia animal, membro torácico, mico-leão-preto, miologia, primatas

## Abstract

*Leontopithecus chrysopygus* (black lion tamarin) is a platyrrhine primate found in the Brazilian Atlantic Forest. This species is threatened by human activities that encroach on its habitat. Studies on *Leontopithecus* myology provide insights into ecological variables and support primate medicine. This study aimed to analyze the anatomy of the forearm muscles of *L. chrysopygus*. For this purpose, six thoracic limbs from five adult male *L. chrysopygus* specimens were dissected. The muscles were described by their attachment points (origin and insertion), and their masses were measured using a precision scale. Functional muscle groups were established for comparison purposes. The positioning and skeletal attachments of the muscles resembled existing descriptions for primates. However, intraspecific variations were noted, such as a recess for the radial nerve between the origin of the tendon of the brachioradial muscle in one-third of the samples. The deep digital flexor muscle had the highest mean mass (2.34 ± 0.43 g), whereas the extensor muscle of the second digit had the lowest mean mass (0.03 ± 0.01 g). The carpal and digital flexor muscles showed a significantly higher mean percentage mass (*p* < 0.05) than the others (58.4 ± 2.7%), possibly due to the strength required for hand grip during locomotion on branches and climbing trees. Further studies on the muscles of other regions of the thoracic limb and other *Leontopithecus* species could enhance our understanding of how these muscles adapt to their lifestyles.

## Resumo

*Leontopithecus chrysopygus* (mico-leão-preto) é um primata platyrrhino encontrado na Mata Atlântica brasileira. Essa espécie está ameaçada devido às atividades antrópicas que invadem seu habitat. Estudos sobre a miologia de *Leontopithecus* fornecem informações valiosas sobre variáveis ecológicas e contribuem para a medicina de primatas. O objetivo deste estudo foi analisar a anatomia dos músculos do antebraço em *L. chrysopygus*. Para isso, foram dissecados seis membros torácicos de cinco espécimes machos adultos de *L. chrysopygus*. Os músculos foram descritos com base em seus pontos de fixação (origem e inserção), e sua massa foi medida com uma balança de precisão. Grupos funcionais musculares foram estabelecidos para fins de comparação. Observou-se que o posicionamento e as fixações esqueléticas dos músculos se assemelham às descrições existentes para primatas. No entanto, foram notadas variações intraespecíficas, como uma reentrância para o nervo radial entre o tendão de origem do músculo braquiorradial em um terço das amostras. A maior massa média foi registrada para o músculo flexor profundo dos dedos (2,34 ± 0,43 g), enquanto a menor massa foi observada no músculo extensor do dedo II (0,03 ± 0,01 g). O grupo de músculos flexores carpais e digitais apresentou uma massa percentual média significativamente maior (p<0,05) em relação aos demais (58,4 ± 2,7%), possivelmente devido à demanda por força na preensão manual durante a locomoção em galhos e escalada de árvores. Estudos envolvendo músculos de outras regiões do membro torácico e de outras espécies do gênero *Leontopithecus* podem ampliar a compreensão da adaptação desses músculos aos estilos de vida dessas espécies.

## Introduction

The order Primates exhibits a wide range of diversity, yet its members share several locomotion-related characteristics. This order includes species with terrestrial and arboreal quadrupedalism, leaping, suspensory movement, and bipedalism. Each locomotion mode has corresponding anatomical adaptations in the trunk and limbs. Arboreal quadrupedalism is the most common locomotion mode among primates, and species that exhibit this mode are found in most evolutionary radiations within the order (Fleagle, 2013).

Locomotor behavior also varies significantly among species in the family Callitrichidae (Platyrrhini) (Youlatos, 2000; Nyakatura & Heymann, 2010; Granatosky, 2018). Some species utilize a vertical clinging and leaping technique, relying heavily on vertical trunks to leap from one to another for movement, foraging, or escaping predators. Other callitrichids prefer horizontal leaping and jump between thin, flexible branches in the canopy, as seen in the *Leontopithecus* genus (lion tamarins) (Garber et al., 2012). The locomotor diversity within the family Callitrichidae forms a continuous spectrum, ranging from vertical leaping specialists to horizontal jumpers, with intermediate degrees of preference. This locomotor diversity is somewhat correlated with variations in feeding behavior (Garber, 1992; Rosenberger, 1992; Porter, 2001).

The skeletal adaptations of primates to different locomotor modes are well-established. However, muscle adaptations are constantly being reviewed and refined. This improves our understanding of the biomechanical and physiological aspects (Fleagle, 2013) and broadens their applications in veterinary medicine, ecology, and species conservation.

Arboreal quadrupedal primates, including lion tamarins, have generalized skeletal morphologies similar to those of their primate ancestors. Their primary challenge is generating propulsion on unstable substrates that are often much smaller than their body size. They overcome this challenge through adaptations that lower their center of gravity toward arboreal support, such as short pelvic and thoracic limbs of comparable length and movement involving more flexed limbs. They also enhance balance through slower locomotion and create a stable base for propulsion with their hands and feet, which have strong grasping abilities (Fleagle, 2013).

*Leontopithecus chrysopygus*, commonly known as the black lion tamarin, is a platyrrhine primate belonging to the family Callitrichidae and subfamily Callitrichinae. It is classified as "endangered" on the IUCN Red List (Rezende et al., 2020). This status is partly due to its endemic distribution in the Brazilian Atlantic Forest, a region with a high human population density (Ankel-Simons, 2007). Another factor threatening the survival of this genus is its highly conspicuous appearance (Ankel-Simons, 2007). For example, *L. chrysopygus* has striking golden fur on its rump and thigh (Kleiman et al., 1988). This species weighs between 540 and 690 grams, has an average head-to-body length of 250–300 millimeters, and has a non-prehensile tail that measures between 360 and 410 millimeters (Valladares-Pádua & Martins, 2016).

*L. chrysopygus* exhibits social behavior characterized by family group formation, task division, and a diet consisting of fruit and insects (Fleagle, 2013). Napier and Napier (1967) classified it as a quadrupedal species that runs, walks, and leaps between terminal branches. It can also descend large trunks, both head-up and head-down (Ankel-Simons, 2007). The species has long hands that appear to be adapted for extractive foraging in specific microhabitats such as bromeliads and tree hollows (Bicca-Marques, 1999).

Anatomical studies on the genus *Leontopithecus* are scarce, as are clinical correlations with the structures described. Marques et al. (1997) conducted a morphometric analysis of the biceps brachii, triceps brachii, and dorsoepitrochlear muscles in *Leontopithecus*. Zdun et al. (2022) examined the forearm musculature of *L. chrysomelas* using imaging techniques. Dickinson et al. (2021) included two specimens of *L. rosalia* in a study on anatomical variations in the forearm muscles of Callitrichidae and Lemuridae. Other recent anatomical studies of this genus have examined the relationship between the gracilis and sartorius muscles in *L. rosalia* (Marques et al., 2006*),* morphometric comparisons of hand size and shape among 31 callitrichine species, including three *Leontopithecus* species (Bicca-Marques, 1999), liver stereology in *Leontopithecus* sp. (Burity et al., 2004), craniometry (Burity et al., 1997), and urogenital system morphology in captive *Leontopithecus* (Pissinatti et al., 2008). However, anatomical knowledge of the *Leontopithecus* genus remains limited.

The lack of anatomical descriptions often leads to the use of other primates or non-primate mammals as references for clinical, surgical, and imaging procedures (Petrucci et al., 2009). This limitation hinders the analysis of how these animals adapt to their environment. Information on forelimb myology in *Leontopithecus* species can provide valuable insights into ecological variables such as behavior, feeding, and reproductive habits, which can ultimately contribute to their conservation. This is particularly important because *Leontopithecus* habitats are increasingly threatened by human activity.

Therefore, this study aimed to elucidate the anatomy of the forearm muscles of *L. chrysopygus*. The findings will improve our understanding of primate morphology and provide an anatomical basis for specialized procedures in primate medicine.

## Material and methods

### Sampling

Six thoracic limbs from black lion tamarins (*Leontopithecus chrysopygus*) were provided by "hidden for review" to "hidden for review," including two limbs from the same individual and all from adult males. The animals died of natural causes and were previously kept under human care. The cadavers were fixed with 10% formaldehyde through intramuscular and intracavitary injections and preserved in the same solution until dissection. As this study did not involve live animals, it was exempt from approval by the Animal Ethics Committee (Law 11,794 of October 8, 2008).

### Dissections

The dissection process involved removing the skin and cleaning the superficial and deep fasciae to facilitate the identification of the forearm muscles. Muscle attachment points, including origin and insertion tendons, were meticulously recorded. After documentation and photographic recording, each muscle was removed by cutting its origin and insertion attachments. Muscle mass was measured using a precision digital scale (± 0.01 g) (Marte®).

For comparison, the forearm muscles were classified into four main functional groups:

1. Carpal and digit extensors (*extensor carpi radialis longus, extensor carpi radialis brevis, extensor digitorum communis, extensor digitorum lateralis, extensor carpi ulnaris, extensor indicis, extensor digiti II, extensor digiti III, and abductor pollicis longus*).
2. Supinators (*brachioradialis and supinator*).
3. Carpal and digit flexors (*flexor carpi radialis, flexor carpi ulnaris, palmaris longus, flexor digitorum superficialis, and flexor digitorum profundus*).
4. Pronators (*pronator teres and pronator quadratus*).

This classification was based on the primary movement for which each muscle acted as an agonist.

### Statistical analysis

After measuring the muscle mass, the arithmetic mean, standard deviation, and coefficient of variation were calculated for each muscle. The mean muscle mass of each functional group was summed, and its percentage relative to the total average forearm muscle mass was determined for group comparison.

Descriptive statistics, including muscle mass, were analyzed using the Shapiro-Wilk normality test, one-way analysis of variance (ANOVA), and post-hoc Tukey's tests to compare the mean percentages across functional groups. These analyses were conducted using GraphPad Prism 8 software with a significance level of *p* < 0.05.

## Results

### Descriptive myology

From a topographical perspective, the forearm muscles are categorized into four groups: superficial craniolateral, deep craniolateral, superficial caudomedial, and deep caudomedial. The superficial muscles were exposed after the skin and superficial fascia were reflected. The deep muscles were made visible after the superficial muscles were reflected, and the deep fascia of the forearm was meticulously cleaned. The craniolateral group contains agonist muscles that promote extension of the carpal and digital joints, as well as supination. In contrast, the caudomedial group comprises primarily agonist muscles involved in carpal and digital flexion and pronation.

#### Superficial craniolateral muscles

**Figure 1:**
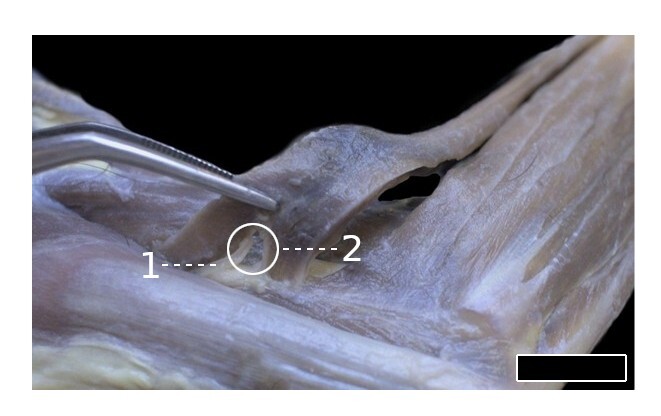
Photomacrograph of the lateral aspect of the right arm and elbow of an adult *Leontopithecus chrysopygus* specimen, showing a branch of the radial nerve (1) passing through a recess (2) located at the origin of the brachioradialis muscle. This recess was observed in one-third of the dissected thoracic limbs. Scale bar: 10 mm.

**Figure 2:**
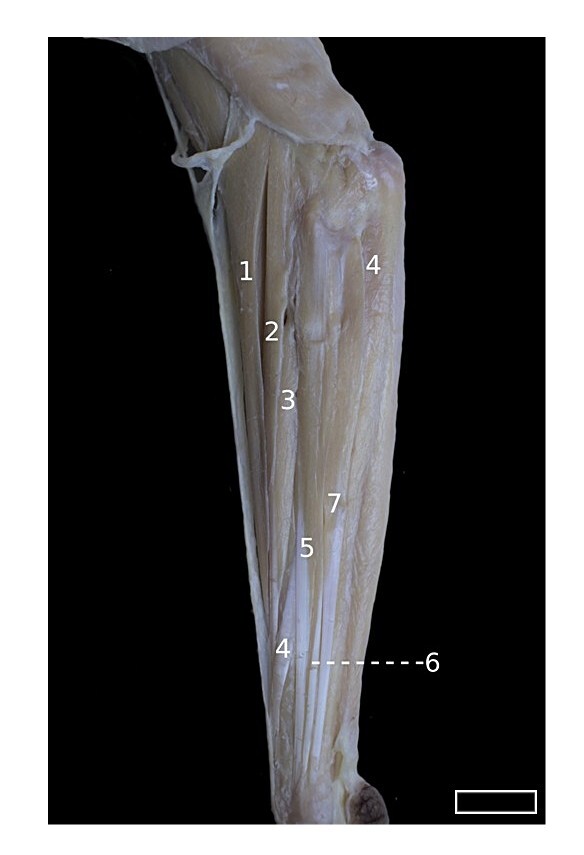
Photomacrograph of the lateral aspect of the forearm of an adult *Leontopithecus chrysopygus* specimen, highlighting the brachioradialis (1), extensor carpi radialis longus (2), extensor carpi radialis brevis (3), abductor pollicis longus (4), extensor digitorum communis (5), extensor digitorum lateralis (6), and extensor carpi ulnaris (7) muscles. Scale bar: 10 mm.

The brachioradialis muscle marks the cranial-superficial border of the extensor muscles of the forearm. This muscle originated from the distal third of the lateral surface of the humeral body, with a second portion at the proximal third of the lateral supracondylar ridge of the humerus. A recess was observed between these two points of origin in two specimens, which allowed passage for the radial nerve. The insertion occurred at the supra-styloid crest of the radius (Figures 1 and 2).

The extensor carpi radialis longus muscle originated from the middle portion of the lateral supracondylar ridge of the humerus. Its insertion occurred via a tendon that formed halfway down the forearm and attached to the second metacarpal. Before reaching its insertion point, the tendon passed deep to the tendon of the abductor pollicis longus muscle and through a compartment of the extensor retinaculum.

The extensor carpi radialis brevis muscle originated from the craniolateral part of the elbow joint capsule and extended to the distal fourth of the lateral supracondylar ridge of the humerus. In the distal forearm, the muscle formed a tendon that passed through a common compartment of the extensor retinaculum, alongside the tendon of the extensor carpi radialis longus, and inserted on the dorsomedial surface of the base of the third metacarpal.

The extensor digitorum communis muscle shared a common origin with the extensor digitorum lateralis on the lateral supracondylar ridge of the humerus. At the beginning of the distal third of the forearm, the muscle divided into three tendons that passed deep to the extensor retinaculum. Upon reaching the dorsum of the hand, these tendons merged to form a dorsal aponeurosis, inserted at the dorsal base of the middle and distal phalanges of digits II, III, IV, and V.

The extensor digitorum lateralis muscle shared a common origin with the extensor digitorum communis. Thus, its origin can be considered indirect because it arose via a shared tendon from the lateral epicondyle of the humerus and the extensor digitorum communis at the lateral supracondylar ridge. It was inserted exclusively on digits IV and V in all specimens.

The extensor carpi ulnaris muscle originated from the lateral epicondyle of the humerus. In the distal third of the forearm, it transitioned from a muscular to a tendinous structure and passed through the most lateral compartment of the extensor retinaculum. Its insertion occurred at the base of the fifth metacarpal.

#### Deep craniolateral muscles

The supinator muscle originated from the lateral epicondyle of the humerus, the lateral collateral ligament of the elbow, and the joint capsule of the elbow. It was inserted on the proximal medial surface and the cranial border of the radius.

The abductor pollicis longus muscle originated from the lateral surfaces of the radius and ulna, and the interosseous membrane, all in the proximal half of the forearm. It was inserted on the first carpal bone and the base of the first metacarpal.

**Figure 3:**
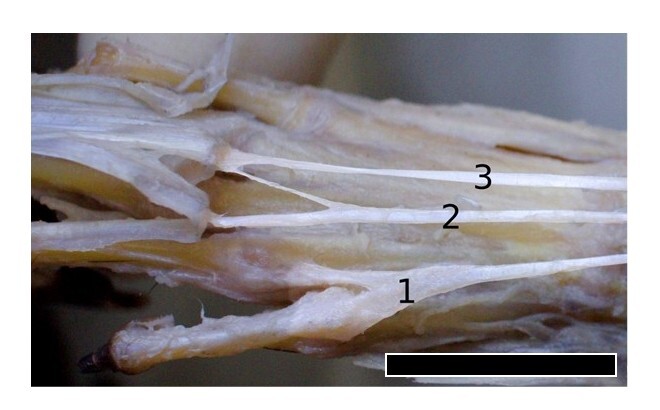
Photomacrograph of the dorsal aspect of the right hand of an adult *Leontopithecus chrysopygus* specimen, highlighting the tendons of the extensor indicis proprius (1), extensor digiti II (2), and extensor digiti III (3) muscles. Scale bar: 10 mm.

The extensor indicis muscle, located laterally to the abductor pollicis longus and medially to the extensor digiti II, originated from the lateral surface of the ulna. It was inserted in the middle dorsal portion of metacarpals I and II, and bifurcated into an aponeurosis that extends towards digits I and II (Figure 3).

The extensor digiti II muscle originated alongside the extensor indicis and extensor digiti III muscles. It was inserted into the phalanges of digit II; however, in two dissected specimens, a thin tendon was also inserted through an aponeurosis shared with the extensor digiti III tendon. The extensor digiti III muscle, which originated adjacent to the extensor digiti II, was inserted into digit III.

#### Superficial caudomedial muscles

**Figure 4:**
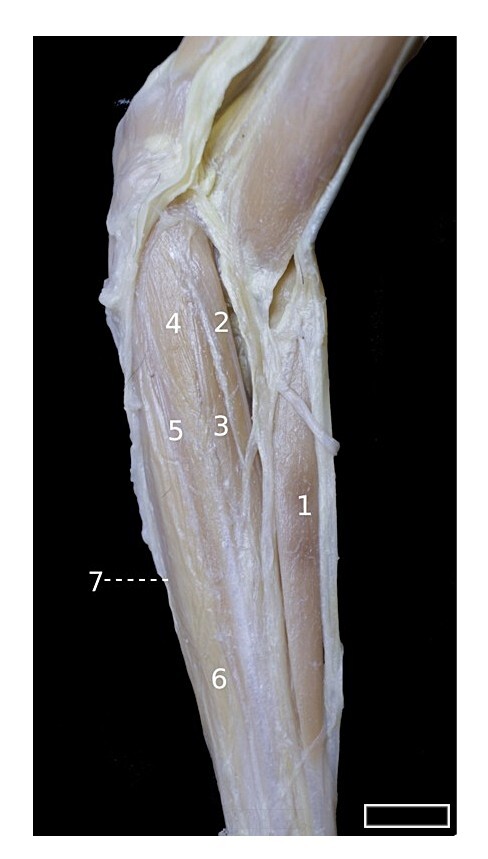
Photomacrograph of the medial aspect of the forearm of an adult *Leontopithecus chrysopygus* specimen, highlighting the brachioradialis (1), pronator teres (2), flexor digitorum profundus (3), flexor carpi radialis (4), palmaris longus (5), flexor digitorum superficialis (6), and flexor carpi ulnaris (7) muscles. Scale bar: 10 mm.

The pronator teres muscle originated from a common tendon shared with the flexor carpi radialis and palmaris longus muscles at the medial epicondyle of the humerus and the medial supracondylar ridge. It was inserted by an aponeurosis on the cranial surface of the radius (Figure 4).

The flexor carpi radialis muscle shared a common tendon of origin with the pronator teres and the palmaris longus at the medial epicondyle of the humerus. It was inserted at the base of the second metacarpal.

The palmaris longus muscle also originated from a common tendon with the pronator teres and flexor carpi radialis at the medial epicondyle of the humerus. Its insertion occurred at the palmar aponeurosis.

The flexor digitorum superficialis muscle originated from a common tendon shared with the humeral head of the flexor digitorum profundus at the medial epicondyle of the humerus. It had four tendons from four muscle bellies, which were inserted into digits II, III, IV, and V.

The flexor carpi ulnaris muscle originated from the medial epicondyle of the humerus and the proximal medial margin of the ulna. It was inserted into the accessory carpal bone.

#### Deep caudomedial muscles

The flexor digitorum profundus muscle originated from the medial epicondyle of the humerus, the interosseous membrane, and the medial surface of the ulna. It had five tendons arising from six heads, which passed together through the carpal canal before inserting at the distal phalanx of each digit.

The pronator quadratus muscle originated from the mid-distal margin of the ulna and was inserted into the mid-distal margin of the radius.

Table 1 summarizes the main origin and insertion characteristics of the forearm muscles in *L. chrysopygus*.

**Table 1:** Primary origin and insertion characteristics of forearm muscles in *L. chrysopygus*.

**Muscle**	**Origin**	**Insertion**
brachioradialis	Body of the humerus and lateral supracondylar ridge of the humerus	styloid process of the radius
extensor carpi radialis longus	Lateral supracondylar ridge of the humerus	Second metacarpal
extensor carpi radialis brevis	Elbow joint capsule and lateral supracondylar ridge of the humerus	Third metacarpal
extensor digitorum communis	Lateral supracondylar ridge of the humerus	digiti II, III, IV and V
extensor digitorum lateralis	Lateral epicondyle of the humerus	digiti IV and V
extensor carpi ulnaris	Lateral epicondyle of the humerus	Fifth metacarpal
extensor indicis proprius	Lateral surface of the ulna	digiti I and II
extensor digiti II	Lateral surface of the ulna	digit II (in two limbs: digiti II and III)
extensor digiti III	Lateral surface of the ulna	digit III
abductor digiti I longus	Lateral surface of the radius, lateral surface of the ulna and on the interosseous membrane	Carpal bone I and metacarpal
supinator	Lateral epicondyle of the humerus, lateral collateral ligament of the elbow and elbow joint capsule	proximal-medial surface and cranial margin of the radius
pronator teres	Medial epicondyle of the humerus and medial supracondylar ridge	cranial surface of the radius
flexor carpi radialis	Medial epicondyle of the humerus	metacarpal II
palmaris longus	Medial epicondyle of the humerus	Palmar aponeurosis
flexor carpi ulnaris	Medial epicondyle of the humerus and proximal-medial margin of the ulna	Accessory carpal bone
flexor digitorum superficialis	Medial epicondyle of the humerus	digiti II, III, IV and V
pronator quadratus	Middle-distal margin of the ulna	middle-distal margin of the radius
flexor digitorum profundus	Medial epicondyle of the humerus, interosseous membrane and medial surface of the ulna	digiti I, II, III, IV, and V

### Quantitative analysis

**Figure 5:**
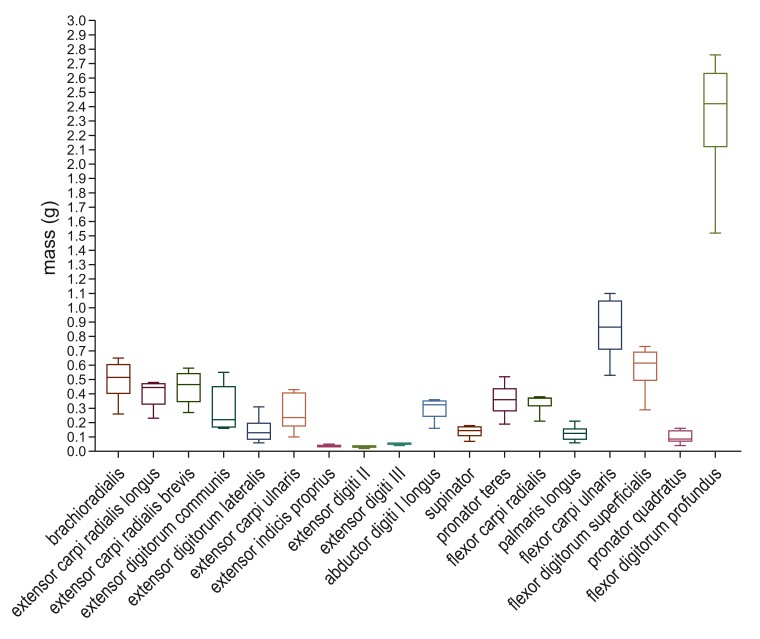
Box-plot graph representing the forearm muscle mass (g) in *Leontopithecus chrysopygus*.

**Figure 6:**
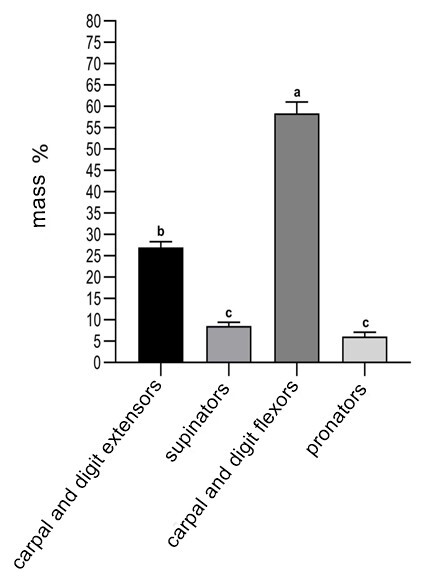
Mean percentage muscle mass ± standard deviation of the four muscle groups identified in the forearm of *Leontopithecus chrysopygus*. Different letters above the bars indicate significant differences (p < 0.05) by Tukey's test*.*

The evaluated sample showed a normal distribution, as indicated by the Shapiro-Wilk test. The combined forearm muscles had an average mass of 7.32 ± 1.52 g. Among the forearm muscles of L. chrysopygus, the flexor digitorum profundus had the highest average mass, 2.34 ± 0.43 g (Table 2 and Figure 5). The flexor muscles of the carpus and digits made up 58.4 ± 2.7% of the total forearm muscle mass, followed by the extensor muscles of the carpus and digits (26.9 ± 1.4%), the supinators (8.6 ± 0.9%), and the pronators (6.1 ± 1.0%) (Figure 6). Analysis of variance showed significant differences in mean percentage mass among the muscle groups (p < 0.05), except between the pronators and supinators.

**Table 2:** Mass values (g), arithmetic mean ± standard deviation (g), and coefficient of variation (CV%) of the forearm muscles from six thoracic limbs of adult male Leontopithecus chrysopygus. The specimens are numbered 1 to 5, and the limb antimers are indicated as R (right) or L (left).

**Muscles**	**1 R**	**2 L**	**2 R**	**3 L**	**4 L**	**5 R**	**Mean ± SD (g)**	**CV (%)**
Brachioradialis (with recess)	0.45	0.56	0.65	0.59	0.26	0.47	0.49 ± 0.14	27.8
Extensor carpi radialis longus	0.36	0.48	0.47	0.44	0.23	0.45	0.41 ± 0.09	23.6
Extensor carpi radialis brevis	0.37	0.58	0.53	0.51	0.27	0.42	0.45 ± 0.12	25.8
Extensor digitorum communis	0.16	0.42	0.55	0.17	0.22	0.22	0.29 ± 0.16	54.7
Extensor digitorum lateralis	0.12	0.14	0.16	0.09	0.06	0.31	0.15 ± 0.08	59.7
Extensor carpi ulnaris	0.40	0.23	0.24	0.43	0.10	0.20	0.27 ± 0.13	41.7
Extensor indicis proprius	0.05	0.04	0.03	0.03	0.04	0.04	0.04 ± 0.01	19.6
Extensor digiti II (and III)	0.04	0.03	0.02	0.04	0.03	0.04	0.03 ± 0.01	24.5
Extensor digiti III	0.04	0.06	0.06	0.05	0.05	0.06	0.05 ± 0.01	15.3
Abductor pollicis longus	0.27	0.32	0.35	0.36	0.16	0.33	0.30 ± 0.07	25.0
Supinator	0.12	0.15	0.18	0.14	0.07	0.17	0.14 ± 0.04	28.7
Pronator teres	0.35	0.37	0.52	0.41	0.19	0.31	0.36 ± 0.11	30.5
Flexor carpi radialis	0.37	0.37	0.35	0.37	0.21	0.38	0.34 ± 0.07	19.1
Palmaris longus	0.06	0.14	0.21	0.11	0.14	0.09	0.13 ± 0.05	41.3
Flexor carpi ulnaris	0.84	1.03	1.10	0.89	0.53	0.77	0.86 ± 0.20	23.5
Flexor digitorum superficialis	0.56	0.61	0.68	0.62	0.29	0.73	0.58 ± 0.16	26.6
Pronator quadratus	0.08	0.14	0.16	0.09	0.04	0.08	0.10 ± 0.04	44.8
Flexor digitorum profundus	2.76	2.59	2.48	2.36	1.52	2.32	2.34 ± 0.43	18.5

## Discussion

### Descriptive myology

The anatomy of the forearm muscles in *L. chrysopygus* demonstrates the broad range of movements that the hands of this species can perform. Although the origin and insertion patterns identified for *L. chrysopygus* closely resemble those of most members of the order Primates, notable differences exist. The brachioradialis muscle has been identified as the primary agonist of supination. In one-third of the dissected limbs, the muscle displayed a recess at its origin that facilitated the passage of the deep branch of the radial nerve. Due to its well-developed structure, the brachioradialis likely plays a critical role in this species' supination, which is essential for branch-to-branch movement (Napier & Napier, 1967).

In most mammals, the extensor carpi radialis is a single muscle (Diogo et al., 2009). In *L. chrysopygus*, the extensor carpi radialis longus and extensor carpi radialis brevis muscles are distinct, as they are in most primates and primate-related taxa such as Scandentia (tree-shrews) and Dermoptera (colugos) (Diogo et al., 2009). Separating these two muscles facilitates carpal extension and some degree of abduction (Aversi‐Ferreira et al., 2010). However, the extensor carpi radialis muscle can appear as a single structure or, occasionally, with additional heads in humans (Tountas & Bergman, 1993).

The common digital extensor muscle of *L. chrysopygus* distributes its tendons to digits II through V, which seems to be a general pattern among platyrrhine primates (Aversi‐Ferreira et al., 2010). However, we found a record of this muscle inserting into digit I in *Callithrix* sp. (Beattie, 1927). The lateral digital extensor muscle of *L. chrysopygus* inserts into digits IV and V, as is the case for most species within the order *Primates* (Aversi‐Ferreira et al., 2010). Nevertheless, Vélez-García et al. (2016) recognized a division of this muscle into two separate extensor muscles for digits IV and V in *Oedipomidas leucopus*. In humans and other primates of the genera *Hylobates*, *Gorilla*, and *Pan*, this muscle typically inserts only into digit V, forming the extensor digiti minimi muscle (Diogo et al., 2009). This difference may be associated with a greater manual dexterity in Hominoidea (Christel, 1993) compared to *L. chrysopygus*. This trait suggests it is an intermediate condition between *L. chrysopygus* and anthropoid primates.

The extensor indicis muscle in *L. chrysopygus* demonstrates insertions into digits I and II, a pattern observed in 80% of dissected specimens of the family *Callitrichidae* (Dickinson et al., 2021). *O*. *leucopus* exhibits the same pattern, though it sometimes inserts into digit III as well (Vélez-García et al., 2021).

In *L. chrysopygus*, the extensor carpi ulnaris muscle originated exclusively from the humerus, which has also been described in other primates such as *Sapajus libidinosus* (Aversi‐Ferreira et al., 2010), *Macaca mulatta* (Howell & Straus, 1933), and *Papio* sp. (Swindler & Wood, 1973). However, in humans, the bony origin develops from both the humerus and the ulna (Netter, 2006).

The supinator muscle has a bony origin on the lateral epicondyle of the humerus, as observed in *O. leucopus* (Vélez-García et al., 2016). However, in some other primates, it also has an additional origin on the ulna, as observed in gibbons (Diogo & Wood, 2012).

Although the pronator teres muscle of *L. chrysopygus* has its bony origin exclusively from the humerus, as in *O. leucopus* (Vélez-García et al., 2016), other primate species, such as *M. mulatta*, also have an ulnar origin (Ackermann, 2003). The presence of an ulnar origin in the supinator and pronator teres muscles in some primates may contribute to increased mechanical advantage or stabilization during supination and pronation, respectively. However, direct functional evidence remains scarce.

The palmaris longus muscle was identified based on anatomical features similar to those observed in other primates and humans. However, its absence has been documented as an anatomical variation in chimpanzees and gorillas (Diogo & Wood, 2012).

The flexor digitorum superficialis muscle of *L. chrysopygus* had a bony origin that was exclusively on the medial epicondyle of the humerus, consistent with descriptions for other primates, such as *O. leucopus* (Vélez-García et al., 2016). Nevertheless, other primates, such as *Pongo pygmaeus*, also exhibit origins on the radius and ulna (Ackermann, 2003).

The flexor digitorum profundus muscle of *L. chrysopygus* revealed three points of origin: the medial epicondyle of the humerus, the interosseous membrane, and the medial surface of the ulna. Descriptions of this muscle in primates are highly variable, ranging from two to six parts (heads) (Stevens et al., 1977; Vélez-García et al., 2016).

### Quantitative analysis

Skeletal muscle mass is a primary variable used in equations that estimate muscle force (Sacks & Roy, 1982). The most straightforward and historically established method for comparing quantitative muscle variables across species is measuring muscle mass in cadavers (Macalister, 1870). Consequently, specific findings were derived from the muscle mass of *L. chrysopygus*.

Despite being the second-smallest group in terms of muscle count, the set of muscles responsible for carpal and digit flexion exhibited the greatest muscle mass proportion among the four examined groups. For a species that moves through arboreal environments and possesses a non-prehensile tail, carpal and digit flexion capacity is crucial for effectively gripping branches and trunks in a vertical orientation (Napier & Napier, 1967). Furthermore, the strength necessary for manipulating fruit and foraging within tree cavities requires significant power from this functional group (Bicca-Marques, 1999).

The carpal and digit extensors had the second-highest muscle mass percentage, reflecting the demand for digit extension movements, including thumb abduction, which increases the surface area for reaching food.

The supinator and pronator muscle groups presented similar average mass percentages, suggesting that the strength required for external rotation of the hand (supination) is counterbalanced by an equivalent force for internal rotation (pronation). Among the pronator muscles, the brachioradialis had the highest mass percentage (23.4%) among those located in the craniolateral compartment. This finding is consistent with observations in other primates, including *S. apella* (30.4%) (Aversi‐Ferreira et al., 2010) and *Papio hamadryas* (24%) (Kikuchi, 2010).

The flexor digitorum profundus muscle exhibited the highest mass percentage among those situated caudomedially, as well as among all forearm muscles. This distinction arises from its insertion on the palmar surface of all digits, which positions it as the primary agonist for digit flexion.

The small sample size and the inclusion of only male specimens prevented comparisons of muscle mass between sexes and antimeres. Marques et al. (1997) reported morphometric differences between males and females, as well as between the right and left antimeres, in some arm muscles of the *Leontopithecus* genus. As suggested by Maple and Finlay (1989), classifying data based on whether animals were kept in captivity or lived in the wild could also result in valuable comparisons. Therefore, future studies comparing the morphometric variables of these muscles across different categories (sex, antimeres, and habitat) may shed light on important issues.

## Conclusions

It can be concluded that the forearm anatomy of *L. chrysopygus* resembles that of other platyrrhine primate species, with slight differences in the origin and insertion points. As expected, *L. chrysopygus* exhibits great similarity in the forearm muscle anatomy to other species in the same family (Callitrichidae), with which it shares postural and locomotor characteristics. Most dissimilarities were observed in relation to the divergent Catarrhini clade, compared to species that also differ from *L. chrysopygus* in posture and locomotion.

This study examined intraspecific anatomical variations, such as a recess for the radial nerve between the origins of the brachioradialis muscle and the emergence of an independent extensor muscle for digit I. The muscles responsible for carpal and digit flexion exhibited a significantly higher average mass percentage than the other groups, reflecting the greater strength required for these movements.
